# Contribution to the discussion of “When should meta‐analysis avoid making hidden normality assumptions?”

**DOI:** 10.1002/bimj.201800189

**Published:** 2018-08-01

**Authors:** Harald Heinzl, Martina Mittlboeck

**Affiliations:** ^1^ Center for Medical Statistics Informatics and Intelligent Systems Medical University of Vienna Vienna Austria

We congratulate Dan Jackson and Ian White for their outstanding work (Jackson & White, 2018) that is pivotal for two reasons. Firstly, the explicit, implicit, and hidden statistical assumptions behind meta‐analyses are carefully presented and discussed; and secondly, the resultant consequences for the applied analysts are considered from a practical viewpoint.

“It has not to be perfect, yet it has to be acceptable” is a stereotyped advice for first‐time reviewers of scientific papers. This advice stresses the fact that there is a zone of reasonableness between excessive meticulousness and inappropriate permissiveness; however, it leaves the definition of range and limits of this zone to individual discretion. The situation seems to be quite similar for meta‐analyses; some violations of their assumptions are minor and may be safely ignored, others are serious and their ignorance would then be a gross error; however, it still remains the responsibility of the individual researcher to decide how to proceed in a given case.

A reasonable, robust, and easy‐to‐apply decision support tool could help to overcome this unsatisfactory situation. Such standardized decision guidance would be particularly beneficial for those applied researchers with only a basic training in statistics. In this regard, Dan Jackson and Ian White suggest the development of a “risk of compromised statistical inference (RoCSI) tool.” Ideally, such a tool will facilitate a fruitful synergy between investigations of statistical assumptions and corresponding content‐related considerations before an appropriate statistical analysis strategy is chosen; this is particularly essential for outlying studies and heterogeneity in general.

- Common outliers in a single study are individual patients, healthy volunteers or laboratory animals and it is usually pretty difficult to figure out the specific reasons why these individual study participants are so widely different from the rest. This should usually not be the case for outlying studies of a meta‐analysis where the study reports enable us, at least in principle, to learn why the results might be deviating. Only thereafter we should consider reaching into our statistical toolbox (nonnormal distribution, downweighting, etc.).
- Dan Jackson and Ian White present a forest plot for the effect of smoking on CRP level (Figure 2, Jackson & White, 2018). A highly heteroscedastic situation $\left(I^{2}=93\%\right)$ is visible and the right statistical path seems predetermined: a random‐effects meta‐analysis. However, just by considering the last two studies only, we may already start feeling uneasy about our right path. These are two huge studies, N = 23,287 and N = 5,452, which differ considerably in their outcomes, β=0.17 [95%CI:0.12,0.22] and β=0.59 [95%CI:0.53,0.64], respectively. How can we reasonably pool two such huge studies without understanding the reasons behind this enormous outcome difference? Similarly, we may be concerned about study 16 that comprises of *N* = 2,577 patients and that is the only one (out of 40) where smoking indicates a decrease of the CRP level.

We conclude by repeating the aforesaid, a meta‐analysis “has not to be perfect, yet it has to be acceptable.” It will remain a challenging task to define “acceptable” both from a subject‐matter and a statistical viewpoint.

## CONFLICT OF INTEREST

The authors have declared no conflict of interest.
